# The role of mesenchymal stem cells in anti-cancer drug resistance and tumour progression

**DOI:** 10.1038/bjc.2012.201

**Published:** 2012-05-17

**Authors:** J M Houthuijzen, L G M Daenen, J M L Roodhart, E E Voest

**Affiliations:** 1 Department of Medical Oncology, University Medical Center Utrecht, Heidelberglaan 100, PO Box 85500, Utrecht 3508 GA, The Netherlands

**Keywords:** mesenchymal stem cells, tumour progression, drug resistance, chemotherapy resistance

## Abstract

It is becoming increasingly clear that the tumour microenvironment has a very important role in tumour progression and drug resistance. Many different cell types within the tumour stroma have an effect on tumour progression either in a positive or in a negative way. Mesenchymal stem cells (MSCs) are a distinct population of cells that have been linked with tumour growth. Mesenchymal stem cells can home to tumours where they modulate the immune system and facilitate tumour growth, angiogenesis and metastasis. Recent studies have shown that MSCs also have an important role in the resistance to various anti-cancer drugs. This mini-review provides an overview of the functional properties of MSCs in tumour progression and drug resistance.

## What are mesenchymal stem cells?

Mesenchymal stem cells (MSCs) are multipotent cells that reside mainly in the bone marrow and in adipose tissue. They are a relatively easy accessible pool of stem cells that have the ability to differentiate into various cell types like osteoblasts, chondrocytes, fibroblasts and adipocytes. Mesenchymal stem cells are characterised as plastic adherent cells that express the markers CD105, CD90, CD73 and lack expression of markers CD45, CD34, CD14, CD11b, CD79*α* or CD19 and HLA-DR (Reagan and Kaplan, 2011). Well established functions of MSCs are suppression of immune responses from cells of the innate and adaptive immunity, homing to damaged tissues and stimulation of angiogenesis (Chen *et al*, 2006). The immune suppressive functions of MSCs are very broad and include regulation via cytokine production, suppression of cytotoxic T-cell functions, inhibition of dendritic cells differentiation and increasing regulatory T cells (Chen *et al*, 2006). It is because of these properties that many research groups are now investigating whether the MSCs can be used in the treatment of transplantation-related diseases like graft versus host disease, autoimmune diseases and as targeted genetically engineered anti-cancer vehicles (Chen *et al*, 2006; Kim *et al*, 2008; Le Blanc *et al*, 2008). Another well described function of MSCs is their ability to migrate to damaged tissues. This unique feature combined with the immune suppressive functions of MSCs has been successfully used to treat myocardial infarction, spinal cord injury, bone injury, damaged kidney and diabetes (Reagan and Kaplan, 2011). Many cytokine and chemokine pathways like SDF-1*α*, TNF-*α*, MMP-2, CXCR4, CXCR1, CXCR2 and CCL8 have been implicated in tissue homing (Ringe *et al*, 2007; Song and Li, 2011). In addition to tissue homing and immune suppression it is also evident that MSCs can stimulate angiogenesis during ischaemia (Schlosser *et al*, 2012) and wound healing (Shome *et al*, 2012). Taken together MSCs fulfil many important tasks under physiological conditions and their unique functions have been successfully used in the treatment of many diseases. However, they have been shown to also affect tumour development.

## The contribution of MSCs to tumour progression

**Figure 1:**
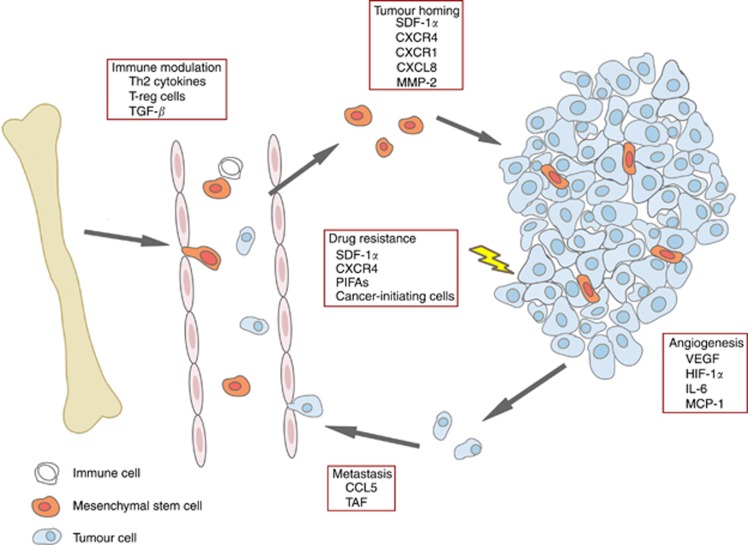
Schematic overview of the role of MSCs in tumour progression and drug resistance. Mesenchymal stem cells can modulate various immune responses allowing tumour cells to evade the immune system. Pathways involved in this process include activation of regulatory T cells, secretion of Th2 cytokines and TGF-*β*. Mesenchymal stem cells can also promote tumour growth by migrating to developing tumours (via SDF-1*α*/CXCR4 signalling, CXCL8 and MMP-2) and subsequent stimulation of proliferation, angiogenesis (via VEGF, IL-6, MCP-1 and HIF-1*α* signalling) and inhibition of apoptosis. Mesenchymal stem cells have also been shown to enhance metastasis directly via CCL5 signalling and indirectly via differentiation into tumour-associated fibroblasts (TAFs). Increasing evidence also shows that MSCs contribute to drug resistance via secretion of PIFAs, activation of SDF-1*α*/CXCR4 signalling or by forming a pool of cancer-initiating cells themselves.

Mesenchymal stem cells are, in addition to other cells in the tumour microenvironment, increasingly identified as an important population of cells that modulate tumour progression and drug sensitivity. The broadly described physiological functions of MSCs in immune suppression have also been linked with tumour progression. It has been shown that in co-cultures of MSCs, breast cancer cells and peripheral blood mononuclear cells MSCs were able to shift the balance of Th1/Th2 cytokines in favour of Th2 cytokines resulting in the ability of the breast cancer cells to evade the immune system (Patel *et al*, 2010). Mesenchymal stem cells can migrate to damaged tissues and as the tumour microenvironment closely resembles damaged tissue MSCs can also to home to developing tumours and promote tumour growth (Loebinger *et al*, 2009). They exert their growth stimulating effects either via paracrine secretion of growth factors and anti-apoptotic factors or by differentiating into tumour-associated fibroblasts, which can enhance tumour growth, metastasis formation and therapy resistance (Hung *et al*, 2007; Mishra *et al*, 2008; Dawson *et al*, 2011) (see Figure 1 for an overview). Evidence for the role of MSCs in metastasis comes from experiments in which non-metastatic breast cancer cells were mixed with MSCs and subsequently injected in mice. Mixing the tumour cells with MSCs increased their potential to metastasise to distant organs compared with mice injected with tumour cells alone. Additional experiments showed that tumour cells induced CCL5 expression in MSCs which in turn enhanced motility and invasion of the cancer cells (Karnoub *et al*, 2007).

In contrast, there are also reports that MSC can also inhibit tumour growth in leukaemia (Ramasamy *et al*, 2007) and hepatoma (Qiao *et al*, 2008) amongst others. Mechanistically, these anti-tumour effects involve downregulation of Akt, beta-catenin, Bcl-2, c-Myc, proliferating cell nuclear antigen and survivin, leading to reduced proliferation, G1 arrest, suppression of oncogenes and increased apoptosis.

These differences in pro- versus anti-tumour effects of MSCs can in part be explained by the tumour types investigated, isolation methods used to obtain the MSCs and numbers of MSCs used. Furthermore, there is a clear difference between the function of MSCs *in vitro* and *in vivo* as was shown for several tumour cell lines including hematopoietic and non-hematopoietic tumour cells. The MSCs exhibited anti-proliferative effects by causing a G1-arrest *in vitro,* however, *in vivo* injection of tumour cells combined with MSCs led to faster tumour growth (Ramasamy *et al*, 2007). These results clearly underscore the challenges ahead of us in investigating how MSCs in one situation block tumour growth and promote growth or metastasis in other situations. In spite of the studies showing a pro-tumourigenic effect, several groups have advocated the use of genetically engineered MSCs as a vehicle to target tumour cells. This approach was successful in models glioma amongst multiple other tumour types (Kim *et al*, 2008). In summary, MSC are pluripotent cells that, once arrived in the tumour microenvironment, can secrete a variety of factors that either positively or negatively influence tumour growth.

## The role of MSCs in drug resistance

Resistance to therapy is one of the major obstacles in the treatment of cancer. Drug resistance can arise within tumour cells because of genetic changes causing for instance increased drug efflux (intrinsic resistance), or it can be the result of the tumour microenvironment protecting tumour cells against treatment (extrinsic resistance). The tumour microenvironment can promote drug resistance in a passive way, by preventing penetration of drugs into the tumour or in an active way by secreting protective cytokines or changing gene transcription within the tumour cells to override the cytotoxic effects of anti-cancer agents (Meads *et al*, 2008). Evidence is now arising that MSCs also have an important role in drug resistance. For a number of haematological malignancies research indicates that MSCs can confer resistance to treatment. Chronic myeloid leukaemia (CML) cells can be protected from imatinib-induced cell death by co-culturing them with MSCs. This protective mechanism was mediated by SDF-1*α* secretion from the MSCs following activation of the CXCR4 pathway in CML cells leading to reduced caspase 3 activity. Inhibition of CXCR4 restored the sensitivity to imatinib (Vianello *et al*, 2010). Furthermore, it was shown that imatinib itself also increased CXCR4 expression on CML cells, indicating that treatment itself can prime tumour cells to such a state that they are more susceptible to the protective effects of MSCs. This increased CXCR4 expression also caused migration of leukaemic cells to the bone marrow resulting in stroma-mediated senescence and chemotherapy resistance (Jin *et al*, 2008). Mesenchymal stem cells are also implicated in drug resistance of chronic lymphocytic leukaemia (CLL). Balakrishnan *et al* (2010) reported that CLL cells can become resistant to the novel drug forodesine. Forodesine induces apoptosis in circulating CLL cells. However, it is unable to effectively kill CLL cells present in the bone marrow where they reside closely to MSCs and other bone marrow cells. Co-cultures of MSCs and CLL cells showed decreased forodesine-induced depletion of ATP and GTP and decreased apoptosis in the CLL cells proving that MSCs are responsible for the reduced effectiveness of the drug (Balakrishnan *et al*, 2010). Mesenchymal stem cells also have a role in drug resistance of acute lymphoblastic leukaemia (ALL). Acute lymphoblastic leukaemia cells express low levels of asparagine synthase (ASNS) and can therefore not supply in their own needs of asparagine. Many patients therefore benefit from asparaginase treatment leading to the depletion of asparagine in the circulation, although resistance to treatment poses a problem. As MSCs express high levels of ASNS and ALL cells grow in close proximity to MSCs it was hypothesised that the MSCs are able to confer resistance to asparaginase treatment by supplying the ALL tumours with asparagine. Iwamoto *et al* (2007) have showed the validity of this hypothesis by demonstrating that co-culturing MSCs with ALL cells decreased the asparaginase-induced cytotoxicity.

Resistance against anti-cancer agents occurs not only in haematological malignancies, MSCs have also been implicated in resistance to chemotherapy of solid tumours. Head and neck squamous cell carcinoma cell lines FaDu and HLaC 78 are more resistant to paclitaxel treatment when co-cultured with bone marrow-derived stem cells (BMSC). The use of a trans-well system indicated that factors that give chemoresistance are secreted by the BMSCs and that no direct cell–cell contact is needed (Scherzed *et al*, 2011). Evidence also exists for a role of MSCs in ovarian cancer therapy resistance. Intraperitoneal chemotherapy is an effective strategy to treat patients with ovarian cancer. Recently, studies have been conducted in which intraperitoneal chemotherapy was combined with hyperthermia in ovarian cancer patients. In contrast with the high success rate of this approach in colorectal cancer, it proved unsuccessful for the treatment of ovarian cancer. Lis *et al* (2011) demonstrated that tumour-associated MSCs are able to protect ovarian cancer cells from the hyperthermia-induced cell death via SDF-1*α*/CXCR4 signalling.

Another mechanism by which MSCs can confer resistance to chemotherapy is by their conversion to cancer-initiating cells or cancer stem cells. Cancer stem cells have the capacity to initiate tumour formation, metastasis and are believed to be highly resistant to anti-cancer drugs and therefore responsible for recurrence. Teng *et al* (2011) has recently shown that targeted methylation of the promoters of two tumour suppressor genes *RasF1A* and *HIC1* in MSCs caused their transformation towards cancer-initiating cells and enabled them to grow in an anchorage-independent manner, formed tumours in nude mice and showed increased resistance to cisplatin treatment. This sheds a new light on the role of MSCs in tumour formation and will require further research if MSCs under physiological conditions by this mechanism can also form a pool of possible cancer-initiating cells. In addition to this the hypothesis has been posed that MSCs can sustain a cancer stem cell niche *in vivo* thereby sustaining malignancy and possibly also drug resistance (Ramasamy *et al*, 2007).

## Mesenchymal stem cells can generate systemic chemotherapy resistance independent of the tumour microenvironment

Recently, we have shown that MSCs can confer chemotherapy resistance in solid tumours in mice. Injection of MSCs i.v. or s.c. at a distant site from the tumour at the time of cisplatin treatment led to complete resistance to the treatment. The ability to induce resistance was unique to MSCs as differentiated progeny were not able to induce resistance, indicating that this feature is lost in the differentiation process. The resistance was mediated by the release of two distinct platinum-induced fatty acids (PIFAs) in response to the cisplatin. The PIFAs that were identified are 12-S-keto-5,8,10-heptadecatrienoic acid (KHT) and 4,7,10,13-hexadecatetraenoic acid (16 : 4(n−3)). 12-S-keto-5,8,10-heptadecatrienoic acid is the oxidised metabolite of 12-S-hydroxy-5,8,10-heptadecatrienoic acid (12-S-HHT) which is a fatty acid that is formed as a by-product in the production of thromboxane A2 synthesis within the arachidonic acid (AA) pathway. Metabolism of AA leads to the production of a series of active fatty acids derivatives also known as eicosanoids including 12-S-HHT and KHT. Very little is known about 16 : 4(n−3), it is an omega-3 fatty acid and it has been described to be produced by some marine algae (Ishihara *et al*, 2000). The PIFAs were able to induce resistance against a broad spectrum of anti-cancer agents and were functional in picomolar concentrations. Systemic treatment of tumour-bearing mice with these PIFAs also showed resistance to chemotherapy, indicating that the presence of the MSCs itself was not needed. The production of these PIFAs was depended on COX-1 and thromboxane synthase, as inhibitors for these enzymes were able to block the formation of resistance (Roodhart *et al*, 2011).

These novel findings show that MSCs can also generate a systemic response to chemotherapy distant from the tumour, rather than initiating a response only from within the tumour microenvironment. Both ways can lead to resistance against various anti-cancer agents. Our data show that the MSCs are activated by platinum-containing therapeutics but protect against a broad range of chemotherapeutics. These MSCs did not home to the tumour or were part of the tumour microenvironment, indicating that the host can also elicit a broader mechanism of resistance reaching beyond the tumour microenvironment. Furthermore, this novel role of MSCs in systemic therapy resistance can possibly be placed in a bigger perspective where MSCs are functioning as guarding cells of the body protecting it from exogenous and cytotoxic agents, backfiring on us in the case of anti-cancer treatment.

It is yet unknown why platinum-containing agents can activate the MSCs. Mesenchymal stem cells express relatively high levels of glutathione (GSH), which is needed to protect these long-lived cells against toxic compounds and reactive oxygen species (Valle-Prieto and Conget, 2010). One hypothesis is that increased cellular GSH can cause a shift in the balance between AA and eicosapentaenoic acid (EPA)-derived eicosanoids in favour of AA metabolism (Rouzer *et al*, 1982). This could explain the increased production of KHT as a downstream metabolite of AA but not the production of 16 : 4(n−3), which is an omega-3 fatty acid and not formed from AA. Arachidonic acid- and EPA-derived eicosanoids counteract in many ways each other’s functions. Arachidonic acid is pro-inflammatory and EPA anti-inflammatory, and a multilevel regulation maintains the balance between the production of these eicosanoids (Whatley *et al*, 1990). It is therefore interesting that in response to platinum-containing therapeutics MSCs produce both fatty acids. Another hypothesis is that platinum-containing drugs elicit some type of stress response leading to the uncoupling of the balance between AA and EPA metabolism leading to the production of both KHT and 16 : 4(n−3). Hypothetically other drugs can activate similar mechanisms in MSCs or other stem cells, either proximal or distal to the tumour leading to activation of systemic resistance-inducing pathways. Identifying these stress signals warrants further studies.

## Clinical relevance of MSCs in tumour progression

Most studies use *in vitro* cultured MSCs or injected supraphysiological concentrations of MSCs, making it difficult to draw conclusions about endogenous levels of MSCs and their role in tumour progression and therapy resistance. Furthermore, clinical data on the role of MSCs in tumour progression is still lacking. Results from Bian *et al* (2009) showed that patients suffering from bone sarcoma have increased numbers of circulating MSCs compared with healthy volunteers. However, within this study no correlations were made between numbers of MSCs present in the blood and therapy response or prognosis (Bian *et al*, 2009). The increased number of circulating MSCs in cancer patients indicates that the presence of a tumour elicits a response causing the MSCs to migrate into the circulation. Interestingly, the presence of these MSCs in the circulation places them in a position where they are easily exposed to relatively high concentrations of anti-cancer drugs like chemotherapy making it more likely that these cells can become activated by the drugs and have a significant role in therapy response. Our data showing that circulating MSCs induce resistance to chemotherapy supports this hypothesis. The relative contribution of resident MSCs in the bone marrow or adipose tissue to drug resistance is yet unknown. It is likely that the differences in the number of circulating MSCs and their potential to respond to anti-cancer drugs between patients determine therapy response rather than total numbers of MSCs.

## Conclusion

A remarkable characteristic of the role of MSCs in tumour progression is its broad versatility and plasticity. Mesenchymal stem cells have been implicated in promoting almost every hallmark of cancer including angiogenesis, metastasis, anti-apoptosis, pro-survival and evasion of immune system. Mesenchymal stem cells can promote tumour growth and drug resistance either via close contact with the tumour cells or via systemic mechanisms involving secreted factors. Targeting MSCs as part of anti-cancer therapy can significantly inhibit tumour growth and metastasis and increase therapy response *in vitro* and in mouse models. As MSCs have such diverse ways to enhance tumour progression, inhibiting these cells in a clinical setting will be challenging. The potent homing of MSCs to tumours is already explored to selectively target tumours. However, it should be kept in mind that these genetically engineered MSCs still contain the numerous intrinsic mechanisms that can promote tumour growth, metastasis and drug resistance indicating that using them as vehicles to target tumours may have severe side effects if not used very carefully.

Despite the enormous amount of data present on the tumour-promoting properties of MSCs there are also reports that MSCs can inhibit tumour growth (see Table 1 for an overview of studies). Perhaps depending on different stimuli the MSCs can also polarise into different states like it has been described for macrophages and more recently also neutrophils. However, what causes the MSCs to become tumour promoting or inhibiting is still unknown. The contradictions between studies could be because of the number of cells used, the source of the MSCs, the cancer types investigated or other components within the tumour microenvironment that will influence the function of the MSCs.

Taken together, MSCs are very important regulators of tumour growth and key modulators of treatment response. This makes these cells an attractive target for therapy, which deserves further fundamental and translational studies.

## Figures and Tables

**Table 1:** MSCs promote or inhibit tumour progression via the following processes

**Process/pathways**	***In vitro vs in vivo***	**Source of MSCs**	**Organism/cell lines used**	**Results**	**Reference**
*Angiogenesis*					
VEGF, HIF-1*α*, IL-6, MCP	*In vitro*	Human bone marrow (BM) derived and murine BM derived	HAEC	Conditioned media from MSCs cultured under hypoxia-contained increased levels of IL-6, VEGF and MCP-1 leading to increased tube formation of HAECs	Hung *et al*, 2007
					
*Metastasis*					
CCL5	*In vivo*	Human BM derived	Nude and NOD-SCID mice with MDA-231-MB tumours	Coimplantation of MSCs increase metastasis potential of weakly metastatic tumour cells. Tumour cells induce secretion of CCL5 from the MSCs leading to increased metastasis	Karnoub *et al*, 2007
TAFs	*In vivo*	Murine BM derived	FVB mice with LA-P2097 tumours and C57bl/6 mice with LLC tumours	Coimplantation of MSCs with tumour cells caused differentiation of MSCs into TAFs. The TAFs persisted in tumour stroma and increased metastasis formation	Dawson *et al*, 2011
					
*Immune modulation*					
Th2 cytokines, TGF-*β* and Treg cells	*In vitro*	Human BM derived	Human blood-derived PBMCs, MDA-231-MB, MCF7, T47D, P815 and K562	MSCs help breast cancer cells to evade the immune system by shifting the balance of Th1/Th2 cytokines towards Th2 cytokines and by increasing the pool of T-regulatory cells	Patel *et al*, 2010
					
*Tissue homing*					
SDF-1*α*/CXCR4 and MMP-2	*In vitro* and *in vivo*	Rat BM derived	*In vivo* model: NOD-SCID mice with PC3 tumours or DUI45 tumours. *In vitro* cell lines: MCF7, PC3, DUI45 and RIF-1	Conditioned media from tumour cells-induced CXCR4 expression in MSCs and reduced MMP-2 expression leading to migration of the MSCs towards the tumours *in vitro* and *in vivo*	Song and Li, 2011
CXCR1 and CXCL8	*In vitro*	Human BM derived	Human MSCs in chemotaxis assay	Migration of MSCs is mediated by CXCR1 and CXCL8 signalling	Ringe *et al*, 2007
					
*Drug resistance*					
SDF-1*α*/CXCR4	*In vitro* and *in vivo*	Human BM derived	*In vivo* model: NOD-SCID mice with BV173 tumours. *In vitro* cell lines: BV173 and primary CML cells	SDF-1*α*/CXCR4 signalling between MSCs and CML cells induces resistance against imatinib *in vitro*. CML cells repopulation in mice was increased when CML cells were pretreated with MSCs and imatinib compared with imatinib alone	Jin *et al*, 2008; Vianello *et al*, 2010
Decreased apoptosis	*In vitro*	Human and murine BM derived	Primary CLL cells from patients co-cultured with MSCs	MSCs protect CLL cells against forodesine-induced apoptosis via decreasing the ATP and GTP depletion normally induced by forodesine	Balakrishnan *et al*, 2010
Asparagine	*In vitro*	Human BM derived	Primary ALL cells from patients	MSCs supply ALL cells with asparagine as a mechanism to evade asparaginase treatment-induced cell death	Iwamoto *et al*, 2007
SDF-1*α*/CXCR4	*In vitro*	Tumour-associated MSCs isolated from ascites and BM-derived MSCs (both human)	SKOV3 and CaOV3 cell lines	MSCs protect ovarian cancer cells from hyperthermia-induced cell death via SDF-1*α*/CXCR4 signalling	Lis *et al*, 2011
Mechanism unknown	*In vitro*	Human BM derived	FaDu and HLaC 78 cell lines	Head and neck squamous cell carcinoma cells co-cultured with MSCs are more resistant to paclitaxel treatment	Scherzed *et al*, 2011
PIFAs	*In vivo*	Human and murine BM derived	Balb/c mice with C26 tumours, C57bl/6 mice with LLC tumours and nude mice with MDA-231-MB tumours	MSCs can induce chemotherapy resistance via the secretion of platinum-induced fatty acids (PIFAs)	Roodhart *et al*, 2011
Cancer-initiating cells	*In vitro* and *in vivo*	Human BM derived	*In vivo* model: nude mice with methylated or control MSCs. *In vitro* cell lines: bone marrow-derived MSCs	Targeted methylation of the promoter of *RasF1A* and *HIC1* transforms MSCs into cancer-initiating cells that grow anchorage independent, form tumours in mice and exhibit cisplatin resistance	Teng *et al*, 2011
					
*Proliferation*					
G1 arrest	*In vitro* and *in vivo*	Human BM derived	*In vivo* model: NOD-SCID mice with BV173 tumours. *In vitro* cell lines: BV173, K562, KG1a, Jurkat, UCH10 and CC3	*In vitro* G1 arrest and decreased prolifation, *in vivo* increased tumour growth	Ramasamy *et al*, 2007
Inhibition of proliferation	*In vitro*	Human foetal dermal derived	H7402 and HepG2	Decrease in beta-catenin, c-myc, Bcl-2, PCNA and survivin leading to decreased proliferation and increased apoptosis	Qiao *et al*, 2008
TAFs	*In vitro* and *in vivo*	Human BM derived	*In vivo* model: nude mice with MDA-231-MB tumours. *In vitro* cell lines: MDA-231-MB, PANC-1 and U87	conditioned media from tumour cells-induced differentiation of MSCs in TAFs. These TAFs promote tumour growth	Mishra *et al*, 2008

Abbreviations: ALL=acute lymphoblastic leukaemia; ATP=adenosine-5′-triphosphate; CCL5=chemokine (C-C motif) ligand 5; CLL=chronic lymphocytic leukaemia; CML=Chronic myeloid leukaemia; CXCL; chemokine (C-X-C motif) ligand; CXCR=chemokine (C-X-C motif) receptor; GTP=guanosine-5′-triphosphate; HAEC=human aortic endothelial cells; HIF-1*α*=hypoxia inducible factor 1 alpha; IL-6=interleukin 6; MCP-1=monocyte chemotactic protein 1; MMP-2=matrix metalloproteinase 2; MSC=mesenchymal stem cell; NOD-SCID=non obese diabetic-severe combined immune deficient; PCNA=proliferating cell nuclear antigen; PIFA=platinum-induced fatty acid; SDF-1*α*=stromal cell derived factor 1 alpha; TAF=tumour-associated fibroblast; VEGF=vascular endothelial growth factor.

## References

[bib1] Balakrishnan K, Burger JA, Quiroga MP, Henneberg M, Ayres ML, Wierda WG, Gandhi V (2010) Influence of bone marrow stromal microenvironment on forodesine-induced responses in CLL primary cells. Blood 116: 1083–1091 20442367 10.1182/blood-2009-10-246199 PMC2938131

[bib2] Bian ZY, Li G, Gan YK, Hao YQ, Xu WT, Tang TT (2009) Increased number of mesenchymal stem cell-like cells in peripheral blood of patients with bone sarcomas. Arch Med Res 40: 163–168 19427966 10.1016/j.arcmed.2009.01.002

[bib3] Chen X, Armstrong MA, Li G (2006) Mesenchymal stem cells in immunoregulation. Immunol Cell Biol. 84: 413–421 16869941 10.1111/j.1440-1711.2006.01458.x

[bib4] Dawson MR, Chae SS, Jain RK, Duda DG (2011) Direct evidence for lineage-dependent effects of bone marrow stromal cells on tumor progression. Am J Cancer Res 1: 144–154 21822499 PMC3150110

[bib5] Hung SC, Pochampally RR, Chen SC, Hsu SC, Prockop DJ (2007) Angiogenic effects of human multipotent stromal cell conditioned medium activate the PI3K-Akt pathway in hypoxic endothelial cells to inhibit apoptosis, increase survival, and stimulate angiogenesis. Stem Cells. 25: 2363–2370 17540857 10.1634/stemcells.2006-0686

[bib6] Ishihara K, Murata M, Kaneniwa M, Saito H, Komatsu W, Shinohara K (2000) Purification of stearidonic acid (18:4(n-3)) and hexadecatetraenoic acid (16:4(n-3)) from algal fatty acid with lipase and medium pressure liquid chromatography. Biosci Biotechnol Biochem 64: 2454–2457 11193415 10.1271/bbb.64.2454

[bib7] Iwamoto S, Mihara K, Downing JR, Pui CH, Campana D (2007) Mesenchymal cells regulate the response of acute lymphoblastic leukemia cells to asparaginase. J Clin Invest 117: 1049–1057 17380207 10.1172/JCI30235 PMC1821067

[bib8] Jin L, Tabe Y, Konoplev S, Xu Y, Leysath CE, Lu H, Kimura S, Ohsaka A, Rios MB, Calvert L, Kantarjian H, Andreeff M, Konopleva M (2008) CXCR4 up-regulation by imatinib induces chronic myelogenous leukemia CML cell migration to bone marrow stroma and promotes survival of quiescent CML cells. Mol Cancer Ther 7: 48–58 18202009 10.1158/1535-7163.MCT-07-0042

[bib9] Karnoub AE, Dash AB, Vo AP, Sullivan A, Brooks MW, Bell GW, Richardson AL, Polyak K, Tubo R, Weinberg RA (2007) Mesenchymal stem cells within tumour stroma promote breast cancer metastasis. Nature 449: 557–563 17914389 10.1038/nature06188

[bib10] Kim SM, Lim JY, Park SI, Jeong CH, Oh JH, Jeong M, Oh W, Park SH, Sung YC, Jeun SS (2008) Gene therapy using TRAIL-secreting human umbilical cord blood-derived mesenchymal stem cells against intracranial glioma. Cancer Res 68: 9614–9623 19047138 10.1158/0008-5472.CAN-08-0451

[bib11] Le Blanc K, Frassoni F, Ball L, Locatelli F, Roelofs H, Lewis I, Lanino E, Sundberg B, Bernardo ME, Remberger M, Dini G, Egeler RM, Bacigalupo A, Fibbe W, Ringdén O (2008) Mesenchymal stem cells for treatment of steroid-resistant, severe, acute graft-versus-host disease: a phase II study. Lancet 371: 1579–1586 18468541 10.1016/S0140-6736(08)60690-X

[bib12] Lis R, Touboul C, Mirshahi P, Ali F, Mathew S, Nolan DJ, Maleki M, Abdalla SA, Raynaud CM, Querleu D, Al-Azwani E, Malek J, Mirshahi M, Rafii A (2011) Tumor associated mesenchymal stem cells protects ovarian cancer cells from hyperthermia through CXCL12. Int J Cancer 128: 715–725 20725999 10.1002/ijc.25619

[bib13] Loebinger MR, Kyrtatos PG, Turmaine M, Price AN, Pankhurst Q, Lythgoe MF, Janes SM (2009) Magnetic resonance imaging of mesenchymal stem cells homing to pulmonary metastases using biocompatible magnetic nanoparticles. Cancer Res 69: 8862–8867 19920196 10.1158/0008-5472.CAN-09-1912 PMC2833408

[bib14] Meads MB, Hazlehurst LA, Dalton WS (2008) The bone marrow microenvironment as a tumor sanctuary and contributor to drug resistance. Clin Cancer Res 14: 2519–2526 18451212 10.1158/1078-0432.CCR-07-2223

[bib15] Mishra PJ, Mishra PJ, Humeniuk R, Medina DJ, Alexe G, Mesirov JP, Ganesan S, Glod JW, Banerjee D (2008) Carcinoma-associated fibroblast-like differentiation of human mesenchymal stem cells. Cancer Res 68: 4331–4339 18519693 10.1158/0008-5472.CAN-08-0943 PMC2725025

[bib16] Patel SA, Meyer JR, Greco SJ, Corcoran KE, Bryan M, Rameshwar P (2010) Mesenchymal stem cells protect breast cancer cells through regulatory T cells: role of mesenchymal stem cell-derived TGF-beta. J Immunol 184: 5885–5894 20382885 10.4049/jimmunol.0903143

[bib17] Qiao L, Xu Z, Zhao T, Zhao Z, Shi M, Zhao RC, Ye L, Zhang X (2008) Suppression of tumorigenesis by human mesenchymal stem cells in a hepatoma model. Cell Res 18: 500–507 18364678 10.1038/cr.2008.40

[bib18] Ramasamy R, Lam EW, Soeiro I, Tisato V, Bonnet D, Dazzi F (2007) Mesenchymal stem cells inhibit proliferation and apoptosis of tumor cells: impact on *in vivo* tumor growth. Leukemia 21: 304–310 17170725 10.1038/sj.leu.2404489

[bib19] Reagan MR, Kaplan DR (2011) Concise review: Mesenchymal stem cell tumor-homing: detection methods in disease model systems. Stem Cells 29: 920–927 21557390 10.1002/stem.645 PMC4581846

[bib20] Ringe J, Strassburg S, Neumann K, Endres M, Notter M, Burmester GR, Kaps C, Sittinger M (2007) Towards in situ tissue repair: human mesenchymal stem cells express chemokine receptors CXCR1, CXCR2 and CCR2, and migrate upon stimulation with CXCL8 but not CCL2. J Cell Biochem 101: 135–146 17295203 10.1002/jcb.21172

[bib21] Roodhart JM, Daenen LG, Stigter EC, Prins HJ, Gerrits J, Houthuijzen JM, Gerritsen MG, Schipper HS. Backer MJ, van Amersfoort M, Vermaat JS, Moerer P, Ishihara K, Kalkhoven E, Beijnen JH, Derksen PW, Medema RH, Martens AC, Brenkman AB, Voest EE (2011) Mesenchymal stem cells induce resistance to chemotherapy through the release of platinum-induced fatty acids. Cancer Cell 20: 370–383 10.1016/j.ccr.2011.08.010 21907927

[bib22] Rouzer CA, Scott WA, Griffith OW, Hamill AL, Cohn ZA (1982) Arachidonic acid metabolism in glutathione-deficient macrophages. Proc Natl Acad Sci USA 79: 1621–1625 6803245 10.1073/pnas.79.5.1621 PMC346027

[bib23] Scherzed A, Hackenberg S, Froelich K, Kessler M, Koehler C, Hagen R, Radeloff A, Friehs G, Kleinsasser N (2011) BMSC enhance the survival of paclitaxel treated squamous cell carcinoma cells *in vitro*. Cancer Biol Ther 11: 349–357 21127403 10.4161/cbt.11.3.14179

[bib24] Schlosser S, Dennler C, Schweizer R, Eberli D, Stein JV, Enzmann V, Giovanoli P, Erni D, Plock JA (2012) Paracrine effects of mesenchymal stem cells enhance vascular regeneration in ischemic murine skin. Microvasc Res 83(3): 267–275 22391452 10.1016/j.mvr.2012.02.011

[bib25] Shome S, Dasgupta PS, Basu S (2012) Dopamine Regulates Mobilization of Mesenchymal Stem Cells during Wound Angiogenesis. PLoS One 7(2): e31682 22355389 10.1371/journal.pone.0031682 PMC3280323

[bib26] Song C, Li G (2011) CXCR4 and matrix metalloproteinase-2 are involved in mesenchymal stromal cell homing and engraftment to tumors. Cytotherapy 13: 549–561 21171825 10.3109/14653249.2010.542457

[bib27] Teng IW, Hou PC, Lee KD, Chu PY, Yeh KT, Jin VX, Tseng MJ, Tsai SJ, Chang YS, Wu CS, Sun HS, Tsai KD, Jeng LB, Nephew KP, Huang TH, Hsiao SH, Leu YW (2011) Targeted methylation of two tumor suppressor genes is sufficient to transform mesenchymal stem cells into cancer stem/initiating cells. Cancer Res 71: 4653–4663 21518779 10.1158/0008-5472.CAN-10-3418

[bib28] Valle-Prieto A, Conget PA (2010) Human mesenchymal stem cells efficiently manage oxidative stress. Stem Cells Dev 19: 1885–1893 20380515 10.1089/scd.2010.0093

[bib29] Vianello F, Villanova F, Tisato V, Lymperi S, Ho KK, Gomes AR, Marin D, Bonnet D, Apperley J, Lam EW, Dazzi F (2010) Bone marrow mesenchymal stromal cells non-selectively protect chronic myeloid leukemia cells from imatinib-induced apoptosis via the CXCR4/CXCL12 axis. Haematologica 95: 1081–1089 20179085 10.3324/haematol.2009.017178 PMC2895031

[bib30] Whatley RE, Zimmerman GA, McIntyre TM, Prescott SM (1990) Lipid metabolism and signal transduction in endothelial cells. Prog Lipid Res 29: 45–63 2128404 10.1016/0163-7827(90)90005-6

